# Action of Curcumin on Glioblastoma Growth: A Systematic Review with Meta-Analysis of Animal Model Studies

**DOI:** 10.3390/biomedicines12020268

**Published:** 2024-01-24

**Authors:** Ângelo Luís, Leonor Amaral, Fernanda Domingues, Luísa Pereira, José Francisco Cascalheira

**Affiliations:** 1 Centro de Investigação em Ciências da Saúde (CICS-UBI), Universidade da Beira Interior, Av. Infante D. Henrique, 6200-506 Covilhã, Portugal; fdomingues@ubi.pt (F.D.); jfcascalheira@ubi.pt (J.F.C.); 2 Departamento de Química, Faculdade de Ciências, Universidade da Beira Interior, Rua Marquês D’Ávila e Bolama, 6201-001 Covilhã, Portugal; 3 Grupo de Revisões Sistemáticas (GRUBI), Faculdade de Ciências da Saúde, Universidade da Beira Interior, Av. Infante D. Henrique, 6200-506 Covilhã, Portugal; lpereira@ubi.pt; 4 Unidade de Saúde Familiar de Santa Joana, Av. de Dom Afonso V, 3810-203 Aveiro, Portugal; leonorpereira.amaral@gmail.com; 5 Departamento de Matemática, Faculdade de Ciências, Universidade da Beira Interior, Rua Marquês D’Ávila e Bolama, 6201-001 Covilhã, Portugal; 6 Centro de Matemática e Aplicações (CMA-UBI), Universidade da Beira Interior, Rua Marquês D’Ávila e Bolama, 6201-001 Covilhã, Portugal

**Keywords:** curcumin, glioma, glioblastoma, animal model, systematic review, meta-analysis

## Abstract

Gliomas are aggressive brain tumors with poor prognosis even after surgical removal and radio-chemotherapy, stressing the urgency to find alternative therapies. Several preclinical studies evaluating the anticancer effect of curcumin in animal models of glioma are reported, but a systematic review with meta-analysis of these studies, considering the different experimental conditions used, has not been made up to this date. A search in different databases (Pubmed, Web of Science, Scopus, and SciELO) following the PRISMA statement was conducted during November 2023 to systematically identify articles assessing the effect of curcumin in murine xenograft models of glioma and identified 15 articles, which were subdivided into 24 studies. Tumor volume before and after treatment with curcumin or vehicle was extracted and the efficacy of curcumin was evaluated by performing a random effects meta-analysis of the data. Publication bias and the impact of different experimental conditions on curcumin efficacy were assessed. Treatment with curcumin decreased tumor volume. Comparing curcumin with control groups, the overall weighted standardized difference in means was −2.079 (95% CI: −2.816 to −1.341; *p*-value < 0.001). The curcumin effect was observed for different animal models, types of glioma cells, administration routes, and curcumin formulations. Publication bias was identified but does not invalidate curcumin’s effectiveness. The findings suggest the potential therapeutic efficacy of curcumin against glioma.

## 1. Introduction

Gliomas are brain tumors with an aggressive phenotype and poor prognosis. The most common and aggressive form of glioma is glioblastoma, which is an IV-grade astrocytoma originating from neural stem cells (NSC), NSC-derived astrocytes, and oligodendrocyte precursor cells (OPC). Human glioblastoma is a highly infiltrative, diffused, and heterogeneous tumor comprising tumor cells and a small fraction of cancer stem cells (CSCs) with high tumorigenic potential and resistance to chemotherapy [1,2]. Despite available treatments involving surgery followed by radiotherapy and chemotherapy, the world’s overall survival remains very low, with a median survival of patients of 15 months after diagnosis [3]. The more common chemotherapeutic options involve the use of alkylating agents, such as temozolomide, nitrosoureas, such as carmustine, and Avastin, which show some limited efficacy, especially in the context of glioblastoma, where the disease is highly aggressive and resistant to treatment [4]. These compounds may show limited effectiveness due to factors such as the blood–brain barrier (BBB), tumor heterogeneity, and the development of resistance over time, leading to a need for alternative or adjunctive therapies to enhance treatment outcomes. In the case of temozolomide, in a phase III clinical trial, the median survival increased from 12.1 months with radiotherapy alone to 14.6 months with radiotherapy plus temozolomide [5]. Therefore, the search for new compounds with potential therapeutic action against gliomas is essential.

Several natural compounds have shown promising results as potential pharmacologic tools against glioma [6,7,8]. Among these compounds is curcumin, a polyphenolic compound produced by turmeric (*Curcuma longa* L., *Zingiberaceae*), which has antioxidant, anti-inflammatory, neuroprotective, and antiproliferative proprieties, making it a potentially useful therapeutic tool against cancer [6,9]. In fact, in multiple preclinical studies, curcumin has shown both preventive—reducing cancer onset in an animal model of colorectal cancer [9]—and potential therapeutic actions against several types of cancer, including colorectal, liver, pancreas, prostate, breast, ovary, and bladder cancers as well as melanoma and lymphoma [6]. Additionally, dietary curcumin, when administered to mice before inoculation with glioma cells, had a preventive effect in reducing tumor onset [10]. Curcumin also shows a chemopreventive effect in ovarian cancer [10]. The chemotherapeutic potential of curcumin was also revealed in a clinical trial where curcumin improved the efficacy of gemcitabine against pancreatic cancer [10].

**Figure 1:**
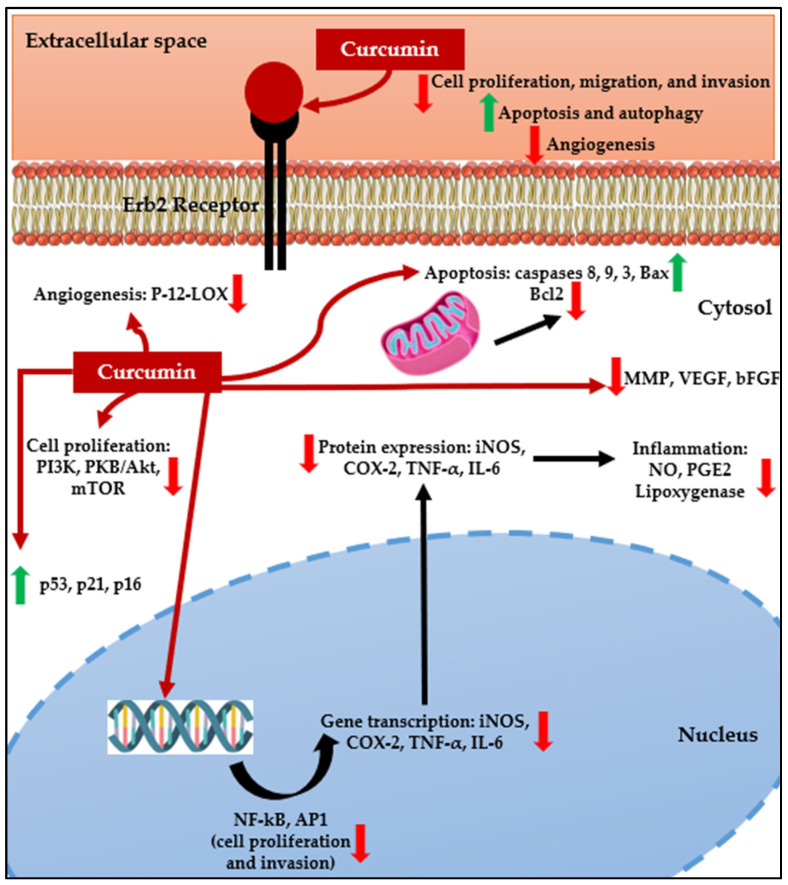
Effects and cellular targets of curcumin on glioblastoma cells (adapted [10,17]).

Several preclinical studies also showed the anticancer action of curcumin against glioma, reducing cell proliferation, migration, and invasion while increasing apoptosis and autophagy of glioma cells and decreasing angiogenesis (Figure 1) [6,11,12]. Furthermore, curcumin lipophilicity allows it to cross the BBB without showing significant toxicity toward normal brain cells, making it a potential pharmacological tool in glioma treatment [11,13]. Curcumin, a pleiotropic molecule, may produce its antiglioma effects either directly or indirectly. Curcumin can directly bind and inhibit several protein targets involved in inflammation (COX and lipoxygenase), cell proliferation (PKC, Src, Erb2), cell survival (Bcl2), and angiogenesis (P-12-LOX) [14]. Additionally, curcumin has been shown to act on several signaling pathways deregulated in cancer, having the ability to (i) reduce the activity of transcription factors NF-kB and AP-1, which are involved in proliferation and invasion [11,15]; (ii) decrease the expression and phosphorylation of PI3K, PKB/Akt, and mTOR, reducing cell proliferation [12,16]; increase levels of caspases 8, 9, and 3 and Bax while decreasing expression of Bcl-2, therefore activating both the extrinsic and intrinsic apoptosis pathways [6,11]; increases expression of p53, p21, and p16, decreasing phosphorylation of Rb and expression of cyclin D, halting the cell cycle [6,11]; and decrease matrix metalloproteinase (MMP), VEGF, and bFGF expression, decreasing invasion and angiogenesis [6,11,17].

The first studies describing an antitumor action of curcumin against glioma cells were reported in the early 2000s, followed by the first reports of the antitumor effect of curcumin on animal models of glioma. Since then, the number of studies reporting an antiglioma action of curcumin using animal models has increased; however, to our knowledge, no systematic review with meta-analysis of these studies has been conducted. Preclinical studies using animal models of diseases play a fundamental role in understanding the mechanisms of disease and testing new therapeutic approaches aiming to improve human health care [18]. Meta-analyses of data from animal studies may help to inform the design of clinical trials or to explain discrepancies between the results of preclinical and clinical trials [19].

The aim of the present work is to perform a systematic review with meta-analysis of the data obtained from studies evaluating the effect of curcumin on tumor growth in animal models of glioma to clarify the therapeutic potential of this natural product. The review was performed in accordance with the Preferred Reported Items for Systematic Reviews and Meta-Analysis (PRISMA) statement.

## 2. Materials and Methods

### 2.1. Search Strategy, Study Selection, and Inclusion Criteria

PubMed, Web of Science, Scopus, and SciELO were the electronic databases searched to identify the papers for this systematic review. The search was performed until 1 November 2023. The string “(curcumin) AND (glioma OR glioblastoma OR astrocytoma)” was used to search the electronic databases using the Boolean operator tools. Duplicate records were eliminated by importing the data into Mendeley Desktop version 1.19.8 (Elsevier, The Netherlands, https://www.mendeley.com/autoupdates/installers/1.19.5, accessed on 17 December 2023). Subsequently, the titles and abstracts of the retrieved publications were screened to filter them for possible inclusion in this systematic review, considering the PRISMA statement [20,21,22]. Furthermore, a check of these articles’ references turned up other publications that may be included. Finally, a careful examination of all the texts that were thought to be relevant was carried out. The search was performed independently by two different authors; a third writer was consulted if there was a dispute. The following requirements were met for a study to be considered for inclusion in this systematic review: it had to show the tumor volume (outcome) at the start and finish of curcumin treatment, include the standard deviation (SD) or standard error of the mean (SEM), and indicate the number of animals in each group (control and treatment). Lastly, it needed to assess how curcumin affects an animal glioma model compared to a control group. The tumor volume was calculated using ImageJ version 1.53t (National Institutes of Health, Bethesda, MD, USA, https://imagej.nih.gov/ij/download.html, accessed on 17 December 2023) when the included publications displayed the data as graphics, pictures, or figures. Moreover, the tumor volume was mostly reported by MRI (magnetic resonance imaging) measurements; however, some papers evaluated the tumor volume by bioluminescence imaging. As other authors found previously [23], the tumor volume is directly proportional to the bioluminescence (R = 0.91), and for that reason, these articles were further included.

### 2.2. Assessment of the Risk of Bias

A 9-item quality checklist that was adapted from the Collaborative Approach to Meta-Analysis and Review of Animal Data in Experimental Studies (CAMARADES) (https://www.ed.ac.uk/clinical-brain-sciences/research/camarades, accessed on 17 December 2023) was used to assess the quality of the methodology of the articles that were included in this systematic review. Using this method, nine factors were assessed [8].

### 2.3. Data Extraction and Synthesis

Two separate authors conducted an analysis of the included studies and extracted data (authors, year of publication, type of cells, animal model, treatment duration, curcumin dose, duration of the treatment, and administration mode) to a Microsoft Excel^®^ file, which facilitated the creation of a database. A third author then compared the two databases, examined the retrieved data, and fixed any discrepancies. Additionally, the baseline and post-treatment tumor volume values were retrieved for the control and intervention groups. The fold increase needed to complete the meta-analysis was determined using these findings.

### 2.4. Statistical Analyses

A weighted standardized difference in means (WSDM) between the pre- and post-treatment mean values of the intervention and control groups was used to evaluate the pooled effect of the curcumin therapy for the examined outcome (tumor volume). The study outlined the parameters for the intervention and control groups, including the number of animals, fold increase, and corresponding SD of the outcome. The statistical analyses were performed using the random effects model and the Comprehensive Meta-Analysis software v2.0 (Biostat, Englewood, NJ, USA, https://www.meta-analysis.com/index.php?cart=BNZ610089737, accessed on 17 December 2023) [24]. To display the impact sizes unique to each research along with a 95% confidence interval (CI), forest plots were created. The degree of discrepancy in the findings of the included studies was measured using the Higgins I^2^ statistic [25], allowing the heterogeneity to be categorized as low (25%), moderate (50%), or high (75%). Subgroup analysis was carried out considering the animal model used, the type of cells, the intervention duration (days), the administration mode, the curcumin mean daily dose (mg/kg/day), the curcumin total dose (mg/kg), and the type of formulation. Using this approach, it was feasible to evaluate how the curcumin impact was affected by various experimental settings and investigate possible causes of variability. The chi^2^ test was also used to see if there was homogeneity among the groupings.

Egger’s regression test [24,26], Duval and Tweedie’s trim and fill method [27,28], and funnel plots [29,30] were employed to assess the potential impact of publication bias. To confirm there is no bias, the trim and fill method creates a funnel plot with the needed imputed studies (shown as red circles) and the observed studies (shown as blue circles), making it also possible to calculate the best estimate of the impartial pooled effect size. Additionally, to evaluate the stability of the results, each research study was removed separately for the sensitivity analysis.

Lastly, the same approach was used to assess the effects of radiation treatment given alone versus radiation treatment coupled with curcumin.

### 2.5. Protocol Registration in PROSPERO

The detailed protocol of this systematic review and meta-analysis was submitted for registration in PROSPERO (https://www.crd.york.ac.uk/prospero/, accessed on 17 December 2023) on 6 December 2023, being attributed the registry number 490884.

## 3. Results

### 3.1. Search and Selection of Studies

**Figure 2:**
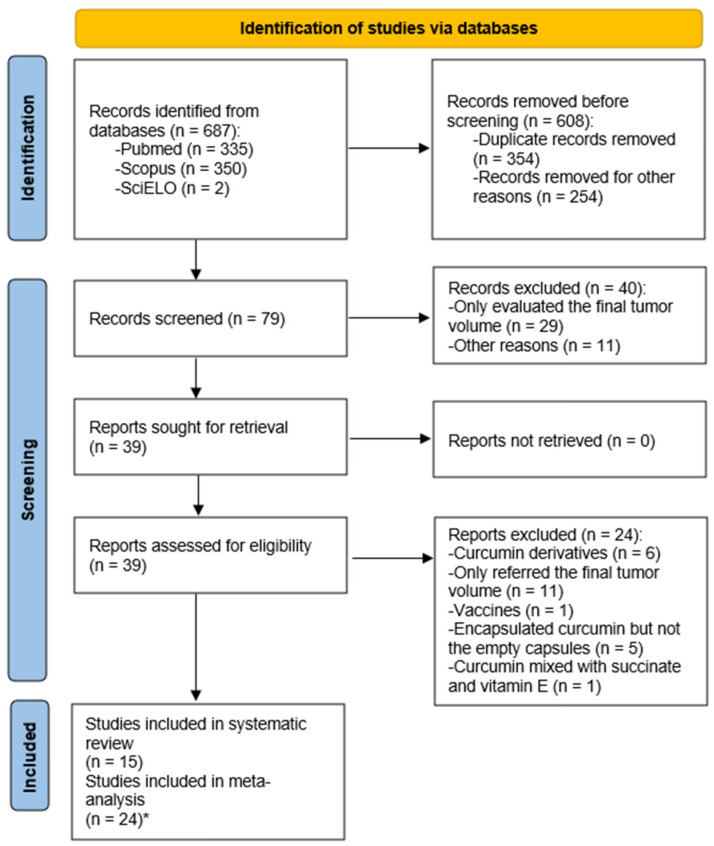
PRISMA flow diagram of the database search, study selection, and articles included in this work. * Indicates the division of the studies explained in the text.

The PRISMA flow diagram of database search, study selection, and articles included in this systematic review with meta-analysis is presented in Figure 2. A total of 687 articles were initially found using the search in the electronic databases. Subsequently, 79 papers remained after the removal of 354 duplicate records and other papers that did not meet the inclusion criteria (254). These papers included studies on the effects of curcumin on glioblastoma in vitro and irregularities in the presentation of the outcome under consideration. Following the full-text analysis, 40 papers were eliminated, mostly for failing to provide the tumor volume measures. After a final examination of the 39 still-eligible papers, 24 were found to be excluded because of issues with research design or result reporting. As such, 15 papers were ultimately included in this systematic review with meta-analysis after the screening procedure.

The work of Wang et al., 2020 [31], evaluated the tumor volume using MRI and bioluminescence. Finally, some works were divided into several studies. The works of He et al., 2020 [32], Jia et al., 2018 [33], and Zheng et al., 2016 [34] were divided into two studies (free curcumin and curcumin micelles). The work of Li et al., 2017 [35], was divided into three studies (three different doses of curcumin). The work of Orunoglu et al., 2017 [36], was divided into four studies (different experimental conditions). The work of Meng et al., 2017 [37], was divided into two studies (different types of cells).

### 3.2. Characteristics of the Included Studies

Table 1 provides a summary of the features of the 24 included studies in this systematic review. The investigations made use of several animal models (heterotopic or orthotopic), as well as human and animal glioblastoma and glioma cell lines. Various curcumin doses, durations of treatment, and administration modes were used. Also, studies employed free curcumin or encapsulated/complexed curcumin. These factors were considered in the subgroup analysis as well.

### 3.3. Risk of Bias Assessment

Table S1 displays the quality scores of the studies as evaluated by the CAMARADES criteria. Only one study by Meng et al., 2017 [37], did not disclose the number of tumor cells implanted in the animals (criterion 2) out of the twenty-four selected research studies, which are peer-reviewed publications (criterion 1). A further six studies [31,32,38,39,40,41] did not address the randomization procedure for assigning tumor-bearing animals to treatment and control groups (criterion 3). Criteria 4 and 5—blinding the outcome evaluation and sample size calculation—were not mentioned in any of the included studies. Most of the articles had quality scores of more than 4, which suggests that they were written correctly overall.

**Table 1:** Characteristics of the 24 included studies in this systematic review with meta-analysis.

Study	Year	Cells	Animals	Outcome Analyzed	Model Used	Intervention	No. of Animals	Dose	Mean Daily Dose (mg/kg/day) Total Dose (mg/kg)	Duration of the Treatment	Administration Mode
Wang et al. [42]	2021	Human glioma LN229	BALB/c-nu/nu nude mice	Tumor volume	Xenografts (subcutaneous, heterotopic)	Control	5	DMEM	-	4 weeks	Intraperitoneal injection
						Curcumin	5	60 mg/kg curcumin/day	60 1260		
Xu et al. [43]	2020	Rat glioma C6	BALB/c nude mice	Tumor volume	Xenografts (subcutaneous, heterotopic)	Blank nanostructured lipid carriers (NLC)	5	-	Does not mention the administration frequency	15 days	Peritumoral injection
						Curcumin–NLC	5	0.2 mg/kg			
						Temozolomide–NLC	5	0.4 mg/kg			
						Curcumin + Temozolomide–NLC	5	0.2 mg/kg + 0.4 mg/kg			
Wang et al. (A) [31]	2020	Rat glioblastoma F98	Male Fischer (F344/NNarl) rats	Tumor volume (MRI) and bioluminescent imaging	Xenografts (brain tumor implantation, orthotopic)	Control	2	2 mL/kg/day of oil (7th to 20th days)	-	14 days	Intraperitoneal injection
						Curcumin	2	120 mg/2 mL/kg/day (7th to 20th days)	120 1680		
Wang et al. (B) [44]	2020	Human U87 glioblastoma cells	Male nude mice	Tumor volume	Xenografts (subcutaneous, heterotopic)	Control	3	Saline	-	13 days	Intraperitoneal injection
						Curcumin	3	60 mg/kg/day	60 780		
He et al. (1) [32]	2020	Mouse glioma GL261	C57 mice	Tumor volume	Xenografts (subcutaneous, heterotopic)	Control	6	Saline	-	9 days	Intraperitoneal injection
						Free curcumin	6	50 mg/kg/every 2 days	27.78 250		
He et al. (2) [32]	2020	Mouse glioma GL261	C57 mice	Tumor volume	Xenografts (subcutaneous, heterotopic)	Blank micelles	6	-	-	9 days	Intraperitoneal injection
						Curcumin micelles	6	50 mg/kg/every 2 days	27.78 250		
Pan et al. [40]	2019	RG2 rat glioma cells	Female adult Wistar rats	Tumor volume	Xenografts (orthotopic)	Empty nanoparticles	24	Not mentioned	Not mentioned	Not mentioned	Not mentioned
						Curcumin nanoparticles	24	Not mentioned			
Jia et al. (1) [33]	2018	Human glioma cell line U251	Female BALB/c nude mice	Tumor volume	Xenografts (orthotopic)	Control	6	PBS	-	7 days	Tail vein injection
						Free curcumin	6	800 µg/day/5 weeks/mice	43.48 304.35		
Jia et al. (2) [33]	2018	Human glioma cell line U251	Female BALB/c nude mice	Tumor volume	Xenografts (orthotopic)	Free exosomes	6	-	-	7 days	Tail vein injection
						Curcumin exosomes	6	800 µg/day/5 weeks/mice	43.48 304.35		
Li et al. (1) [35]	2017	Human glioblastoma U87	Male SCID mice	Tumor volume	Xenografts (subcutaneous, heterotopic)	Control	3	Polyvinylpyrrolidone (PVP)	-	14 days	Oral administration
						Curcumin–PVP	3	30 mg/kg/day	30 420		
Li et al. (2) [35]	2017	Human glioblastoma U87	Male SCID mice	Tumor volume	Xenografts (subcutaneous, heterotopic)	Control	3	PVP	-	14 days	Oral administration
						Curcumin–PVP	3	60 mg/kg/day	60 840		
Li et al. (3) [35]	2017	Human glioblastoma U87	Male SCID mice	Tumor volume	Xenografts (subcutaneous, heterotopic)	Control	3	PVP	-	14 days	Oral administration
						Curcumin–PVP	3	120 mg/kg/day	120 1680		
Singh et al. [41]	2016	Human glioblastoma U87	Male athymic mice (CAnN.Cg-Foxn1nu/Crl)	Bioluminescence	Xenografts (orthotopic)	Control	3	F-127/day	-	21 days	Intravenous injection
						Theranostic photonic nanoparticles–Curcumin	3	0.2 mg/kg curcumin-F-127/day	0.2 4.2		
Orunoglu et al. (1) [36]	2017	RG2 rat glioma cells	Female Wistar rats	Tumor volume	Xenografts (orthotopic)	Control	6	-	-	1 day	Intratumoral
						Curcumin	6	6.7 × 10^−4^ mg/kg/once (25 µM in 20 µL)	0.00067 0.00067		
Orunoglu et al. (2) [36]	2017	RG2 rat glioma cells	Female Wistar rats	Tumor volume	Xenografts (orthotopic)	Control	6	-	-	1 day	Intravenous
						Curcumin	6	6.7 × 10^−4^ mg/kg/once (25 µM in 20 µL)	0.00067 0.00067		
Orunoglu et al. (3) [36]	2017	RG2 rat glioma cells	Female Wistar rats	Tumor volume	Xenografts (orthotopic)	Nanoparticles	6	-	-	1 day	Intratumoral
						Nanoparticles + Curcumin	6	6.7 × 10^−4^ mg/kg/once (25 µM in 20 µL)	0.00067 0.00067		
Orunoglu et al. (4) [36]	2017	RG2 rat glioma cells	Female Wistar rats	Tumor volume	Xenografts (orthotopic)	Nanoparticles	6	-	-	1 day	Intravenous
						Nanoparticles + Curcumin	6	6.7 × 10^−4^ mg/kg/once (25 µM in 20 µL)	0.00067 0.00067		
Meng et al. (1) [37]	2017	Human glioma cell line LN229	Female nude mice	Bioluminescence	Xenografts (orthotopic)	Control	3	DMSO	-	15 days	Intraperitoneal injection
						Curcumin	3	60 mg/kg/day	60 900		
						Radiation	3	18 Gy/once	-		
						Curcumin + Radiation	3	60 mg/kg/day + 18 Gy/once	60 900		
Meng et al. (2) [37]	2017	Human glioma cell line U251	Female nude mice	Bioluminescence	Xenografts (orthotopic)	Control	3	DMSO	-	15 days	Intraperitoneal injection
						Curcumin	3	60 mg/kg/day	60 900		
						Radiation	3	18 Gy/once	-		
						Curcumin + Radiation	3	60 mg/kg/day + 18 Gy/once	60 900		
Zheng et al. (1) [34]	2016	Rat glioma C6	Female nude BALB/c mice	Tumor volume	Xenografts (subcutaneous, heterotopic)	Control	5	Saline	Does not mention the administration frequency	Not mentioned	Intravenous injection
						Curcumin	5	50 mg/kg			
Zheng et al. (2) [34]	2016	Rat glioma C6	Female nude BALB/c mice	Tumor volume	Xenografts (subcutaneous, heterotopic)	Empty micelles	5	-	Does not mention the administration frequency	Not mentioned	Intravenous injection
						Curcumin micelles	5	50 mg/kg			
Yin et al. [45]	2014	Human glioblastoma U87MG cells	nu/nu athymic BALB/c mice	Tumor volume	Xenografts (subcutaneous, heterotopic)	Control	6	Not mentioned	Not mentioned	14 days	Intraperitoneal injection
						Curcumin	6				
						Temozolomide	6				
						Curcumin + Temozolomide	6				
Perry et al. [39]	2010	Human glioblastoma U87MG cells	Athymic female mice (Crl:CD-1 *nu*BR)	Tumor volume	Xenografts (orthotopic)	Control	7	Vehicle	-	26 days	Intraperitoneal injection
						Curcumin	7	60 mg/kg/day	60 1560		
Aoki et al. [38]	2007	Human glioblastoma U87MG cells	Adult nude mice	Tumor volume	Xenografts (subcutaneous, heterotopic)	Control	5	-	-	7 days	Intratumoral injection
						Curcumin	5	100 mg/kg/20 µL in DMSO/PBS/once	100 700		

The letters within brackets indicates different studies by the same first author and numbers within brackets indicates the division of the studies.

### 3.4. Effects of Curcumin on Glioma

**Figure 3:**
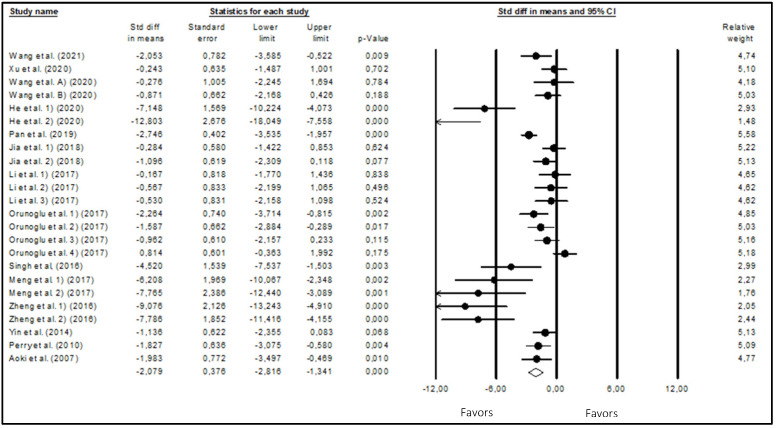
Forest plot of comparisons of the effects of curcumin on glioma growth. Heterogeneity: Tau^2^ = 2.375; chi^2^ = 115.628; df = 23; *p*-value < 0.0001; I^2^ = 80.109. Test for overall effect: Z = −5.523 (*p*-value < 0.0001) [30,31,32,33,34,35,36,37,38,39,40,41,42,43,44].

The meta-analysis results of the effects of curcumin on glioblastoma growth obtained using the random effects model are presented in the Forest plot of Figure 3, with the tumor volume being the outcome analyzed (fold increase from day 1). With 304 animals in the 24 included studies, curcumin was shown to significantly reduce (*p*-value < 0.001) the tumor volume (WSDM = −2.079; 95% CI: −2.816 to −1.341). These findings suggest that curcumin may have antiglioma growth properties. There was a high degree of heterogeneity (I^2^ = 80.109%) across the studies that were considered.

### 3.5. Subgroup and Sensitivity Analyses

To ascertain the impact of the animal model employed, the type of cells, the duration of the intervention, the mode of administration, the mean daily dose, the total dose, and the formulation on the effects of curcumin on glioblastoma development, a subgroup analysis was carried out (Table 2).

With respect to the animal model used, a significant reduction (*p*-value < 0.05) in tumor volume was observed for both heterotopic and orthotopic models. The observed heterogeneity was not explained by the animal model (chi^2^ = 0.549; *p*-value = 0.459).

Concerning the type of cells inoculated, a significant reduction (*p*-value < 0.05) in tumor growth was observed for all types except for rat glioblastoma cells. The observed heterogeneity may be explained by the type of cells on which the studies were performed (chi^2^ = 20.333; *p*-value < 0.0001).

Considering the intervention duration, all studied intervals of intervention duration led to a significant reduction (*p*-value < 0.05) in tumor volume. Furthermore, the length of the intervention was shown to be a significant contributor to the observed heterogeneity (chi^2^ = 9.599; *p*-value = 0.022).

The administration mode did not contribute to the observed heterogeneity (chi^2^ = 5.786; *p*-value = 0.328), and only for intraperitoneal and intravenous (via tail vein) administration was a significant reduction (*p*-value < 0.05) in tumor volume observed.

Regarding the mean daily dose and the total dose, only for moderate doses (20.1–60 mg/kg/day and 200–900 mg/kg) was a significant reduction (*p*-value < 0.05) in tumor volume observed. Neither of these two variables contributed to the heterogeneity. The same was observed for the total curcumin dose.

When curcumin was administered in its free form or encapsulated/complexed, a significant reduction (*p*-value < 0.05) in glioblastoma volume was observed, but once again, the formulation did not contribute to the heterogeneity.

**Figure 4:**
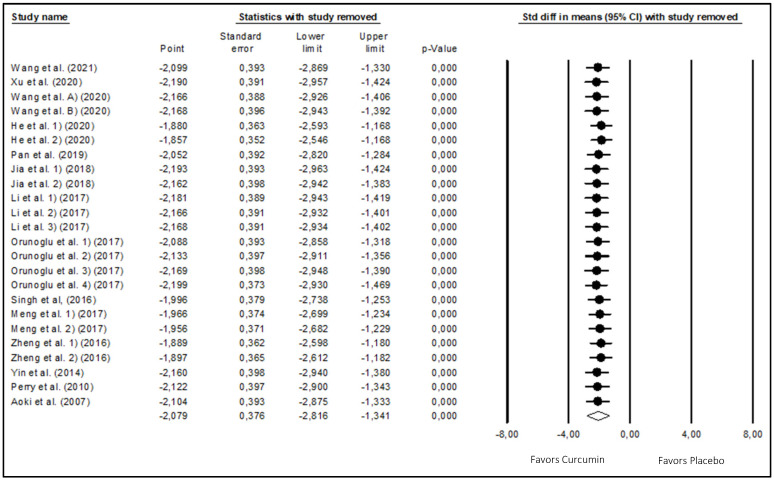
Results of sensitivity analysis [30,31,32,33,34,35,36,37,38,39,40,41,42,43,44].

To investigate how the outcomes might alter if one or more studies had not been included in the meta-analysis, a sensitivity analysis was conducted. The results showed that the pooled effects of curcumin on glioma development were not significantly affected by the exclusion of one or a few trials (Figure 4). Overall, the sensitivity analysis proved that the conclusions of this meta-analysis were legitimate.

### 3.6. Publication Bias

Egger’s regression test was first used to investigate the existence of publication bias (Table 3). The findings (*p*-value = 0.002) support the existence of publication bias.

**Figure 5:**
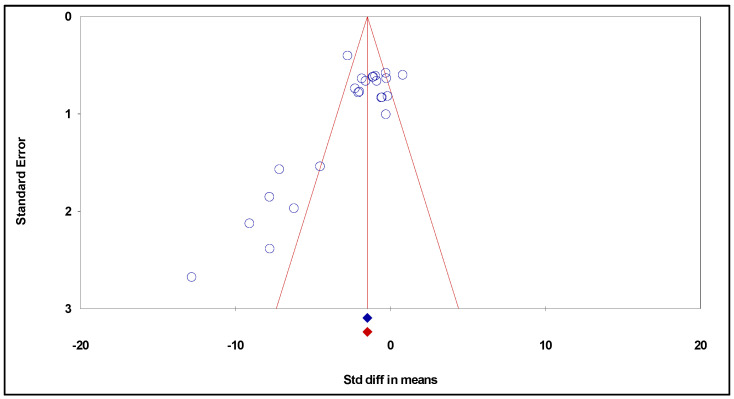
Funnel plot of standard error by standardized difference in means (publication bias tests) of the effects of curcumin on glioma growth.

In addition, the trim and fill approach was taken into consideration while creating the funnel plot (Figure 5). However, this method did not show the necessity to impute additional studies to correct for the lack of bias.

### 3.7. Effects of Radiation Combined with Curcumin on Glioma

**Figure 6:**
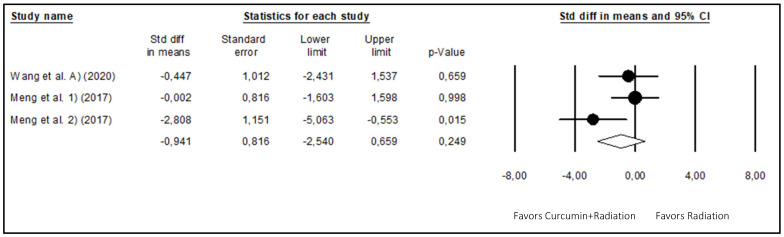
Forest plot comparing the effects of curcumin combined with radiation to radiation alone on glioma growth. Heterogeneity: Tau^2^ = 1.028; chi^2^ = 4.122; df = 2; *p*-value = 0.127; I^2^ = 51.481%. Test for overall effect: Z = −1.153 (*p*-value = 0.249) [30,36].

The effects of radiation treatment given alone versus radiation treatment coupled with curcumin were also meta-analyzed (Figure 6) using the random effects model to identify a potential cumulative action between curcumin and radiation. The results of this meta-analysis, which included three studies (16 animals), showed that the combination of curcumin with radiation did not show a further significant reduction (*p*-value = 0.249) in glioma volume (WSDM = −0.941; 95% CI: −2.540 to 0.659), presenting moderate heterogeneity (I^2^ = 51.481%).

Sensitivity analysis was also carried out to investigate the implications of excluding one or more papers from this meta-analysis (Figure S1). The robustness of these findings was demonstrated by the meta-analysis, which did not alter when comparing the effects of radiation alone against the administration of curcumin together with radiation.

Egger’s regression test (Table S2) was also used to examine the possibility of publication bias, and the findings did not support this theory (*p*-value > 0.05). A funnel plot (Figure S2) was also created by taking the trim and fill approach into consideration. This showed that some studies would be required to establish an absence of publication bias.

## 4. Discussion

Chemoprevention, or the use of harmless natural or synthetic substances to interfere with the progression of carcinogenesis at an early stage, has become a viable and practical medical strategy for lowering the risk of cancer. Numerous plant parts referred to as “phytochemicals” have been shown to have significant chemopreventive qualities. One of the most well-studied and precisely identified chemopreventive phytochemicals is curcumin. Therefore, the effect of curcumin on glioblastoma development in animal model studies was reported in this systematic review with meta-analysis [9].

The present systematic review with meta-analysis involved 15 publications, subdivided into 24 studies, enrolling 304 animals. A significant overall reduction in tumor growth was found after the treatment of murine xenograft models of glioma with curcumin. This antitumor effect of curcumin was observed for different tumor models, cancer cell types, intervention durations, and formulations. When compared to radiation alone, the combination of curcumin plus radiation therapy did not result in any further tumor volume reduction. Since most studies reported the starting and final tumor volumes or the fold of increase in tumor volume, along with the related SD or SEM, the fold increase from the initial volume prior to treatment was selected as the outcome in the current meta-analysis.

When reviewing multiple studies on a subject like curcumin’s effects on cancer, discrepancies or differences might arise due to several factors, namely, dosage and duration (variations in the dosage of curcumin administered or the duration of treatment could lead to differing outcomes), cell lines or models (variations in the cell lines or animal models used across studies might yield different responses due to inherent differences in cell behavior or tumor microenvironments), curcumin formulation and bioavailability (different formulations or methods of delivering curcumin might affect its bioavailability, impacting its effectiveness, and variations in the purity and source of curcumin could also lead to different results), context-specific effects (the complexity of cancer biology and the specific molecular characteristics of different cancer types might result in context-specific responses to curcumin, and its effects on different cancer types may vary due to their unique genetic makeup and signaling pathways), outcome measures (differences in the endpoints assessed across studies could lead to discrepancies), experimental controls (variability in the control groups, treatment protocols, or experimental conditions might impact the interpretation of results), and publication bias (publication bias might lead to the preferential publication of studies with positive results, potentially skewing the overall perception of curcumin’s effects).

Starting from the early 2000s, several preclinical studies reporting an antiglioma effect of curcumin both in vitro, using glioma cells, and in vivo, using animal xenograft models [38,39,46,47,48,49], have been described and some recent reviews [6,12] and one systematic review has been published [11]. This review analyzed 19 in vitro and 5 in vivo studies, indicating that curcumin decreased glioblastoma cell proliferation and viability by acting on multiple pathways inducing a decrease in prosurvival proteins such as NF-kB, AP-1, and PI3K, as well as upregulating proteins involved in apoptotic pathways such as p21, p53, and caspase 3 [11]. The authors concluded that curcumin inhibits proliferation and induces apoptosis and differentiation of glioma-initiating stem cells, and its ability to target multiple signaling pathways involved in cell survival and cell death makes it a potential therapeutic agent [11]. However, to our knowledge, no systematic review with meta-analysis evaluating the antitumor effect of curcumin in glioma-bearing animal models has been conducted, so it has now been performed in the present work.

In this work, subgroup analysis was performed to evaluate the impact of different experimental conditions on the outcome. The results showed a significant reduction in tumor volume induced by curcumin in both orthotopic intracranial and heterotopic subcutaneous xenografts. Furthermore, there was no significant variation in curcumin effect between tumor models.

Regarding the type of cancer cells inoculated, a significant curcumin antitumor effect was observed for human glioblastoma, mouse glioma, and rat glioma cells. No significant effect of curcumin was observed for rat glioblastoma cells; however, the reduced number of studies using this cell type might explain the lack of observation of curcumin effect. Nevertheless, the cell type showed to be a significant source of heterogeneity between studies. The discrepancy in the observed reduction in tumor growth, specifically in rat glioblastoma cells, compared to other types could be attributed to other factors beyond just the reduced number of studies. Rat glioblastoma cells might respond differently to curcumin compared to human or other animal models due to species-specific genetic, metabolic, or microenvironmental differences. Rat glioblastoma cells might possess unique genetic or phenotypic characteristics that render them less responsive to curcumin’s antitumor effects. Tumor microenvironment differences in rat glioblastoma cells could impact their response to curcumin. Factors like vasculature, immune cell infiltration, or stromal interactions might influence curcumin’s efficacy differently in rat glioblastoma models compared to other types.

Considering the curcumin administration mode, only when curcumin was administered intraperitoneally or intravenously was a significant reduction in tumor volume induced by curcumin observed. However, the lack of observation of the curcumin effect for intratumoral, peritumoral, or oral administration might be due to the reduced number of studies—respectively, three, one, and three—using this administration mode. In fact, there was no significant variation between different modes of curcumin administration. The discrepancy in observed tumor reduction based on different administration routes of curcumin (intraperitoneal, intravenous, intratumoral, and peritumoral) could be unexpected and might not solely be attributed to the reduced number of studies in specific administration methods. Intratumoral or peritumoral administration might lead to a more localized concentration of curcumin directly within or around the tumor site. However, in the case of intratumoral and peritumoral administration, the lack of significance of the curcumin effect might result from the very low doses of curcumin (0.00067 mg/kg) applied in two [36] of the three studies using intratumoral administration, and in the sole study [43] using peritumoral delivery (0.2 mg/kg), which produced small or negligible effects. Otherwise, achieving consistent distribution throughout the entire tumor mass might be challenging, potentially limiting its overall efficacy compared to systemic routes. Intratumoral or peritumoral administration might result in different pharmacokinetics and bioavailability profiles compared to systemic routes. Factors such as absorption, metabolism, and clearance rates could differ, impacting the effectiveness of curcumin within the tumor microenvironment. The ability of curcumin to penetrate the tumor cells or reach deeper regions of the tumor mass might vary based on the administration route. Systemic routes like intravenous or intraperitoneal delivery might allow better access to different regions of the tumor compared to direct injections.

The subgroup analysis for different durations of treatment with curcumin showed a significant reduction in tumor volume induced by curcumin when it was applied for 1–10, 11–20, and 21–28 days, a higher effect being observed for the 21–28 days treatment duration. In fact, the duration of treatment was revealed to be a source of heterogeneity between studies. Concerning the mean daily dose of curcumin, a significant reduction in glioma volume was observed only when intermediate doses (20.1–60 mg/kg/day) were applied. The absence of observation of a significant effect of curcumin for higher daily doses was probably because only two studies used mean daily doses higher than 60 mg/kg/day. However, the mean daily dose of curcumin did not contribute significantly to the heterogeneity of the results. Similarly, a significant reduction in tumor volume was only observed for intermediate (200–900 mg/kg) total doses of curcumin. Again, the lack of observation of the significant effect of curcumin for higher total doses might be explained by the reduced number of studies using total doses of curcumin higher than 900 mg/kg. The total dose of curcumin was not a significant source of the heterogeneity.

The safety profile of curcumin, especially at varying doses and prolonged administration, is a crucial aspect to consider in any study or clinical application. Considering the studies analyzed in the present work, which applied higher total doses of curcumin for prolonged periods, specifically 1560 mg/kg for 26 days [39] and 180 mg/kg for 14 days [35], no obvious side effects of curcumin treatment were observed throughout the duration of the studies, showing the animals’ good activity with normal food and water intake and demonstrating normal weight gain, indicating that mice tolerated the treatment well [35,39].

The low bioavailability of curcumin is a significant challenge in harnessing its full therapeutic potential. Regarding the curcumin formulation, both free and non-free (encapsulated, complexed, and incorporated in lipid micelles) produced a significant reduction in tumor volume. There was no significant variation between different formulations of curcumin, and the curcumin formulation did not account for a significant proportion of the heterogeneity. This result is somehow surprising since the use of curcumin encapsulated in nanoparticles or incorporated in lipid micelles is intended to increase curcumin solubility, bioavailability, and brain delivery [11,12]. The similar results observed for both free and non-free curcumin might be attributed to the high lipophilicity and the ability to cross the BBB of free curcumin [6]. While various approaches have been explored to enhance its bioavailability, the surprising nature of the findings might necessitate a deeper exploration and novel strategies. Although using nanosized curcumin particles or encapsulation in liposomes or nanoparticles can improve solubility and absorption, challenges remain in large-scale production and stability. One hypothesis to enhance the bioavailability of curcumin may be related to piperine co-administration (piperine, found in black pepper, is known to enhance curcumin absorption; however, its effectiveness might vary among individuals, and long-term safety is a concern) [50]. Strategies to modify the gut microbiome for improved curcumin absorption might also be an interesting possibility. Exploring combinations with other natural compounds or pharmaceutical agents that could synergistically enhance curcumin’s bioavailability or activity can also be considered.

The effect of a combined treatment of glioma-bearing animal xenografts with curcumin and radiation was evaluated in the present meta-analysis. The overall effect of the combined treatment on tumor growth was not significantly different from the effect of radiation alone, with only one study showing a significant decrease in tumor volume produced by the combined application of curcumin plus radiation when compared with radiation alone. However, the absence of observation of a significant effect might be due to the reduced number of studies, since only three studies were published and included in the meta-analysis that described the combined effect of curcumin plus radiation. In fact, in one study [31], combined treatment with curcumin plus radiation produced a significantly higher (*p*-value < 0.05) percentage of survival after 60 days of tumor implantation when compared with radiation alone, even though a significant reduction (*p*-value < 0.05) in tumor volume was not observed, probably because of the reduced number of animals (n = 2) used in the experiments evaluating tumor growth. Regarding the two other studies [37], a significant reduction (*p*-value < 0.05) in tumor volume was observed in mice treated with curcumin plus radiation when compared with radiation alone, but only for mice implanted with U251, not LN229 human glioblastoma cell lines, suggesting that the efficacy of combined curcumin plus radiation treatment might depend on the type of glioblastoma cell line. Otherwise, the timing and sequence of curcumin administration in relation to radiation therapy might influence their interaction. If curcumin is administered at a time that does not synchronize well with radiation exposure, synergistic effects might not be fully realized. Glioma cells might have variable responses to radiation, with some subsets being more or less sensitive.

In the present meta-analysis, even after subgrouping for site of cell tumor inoculation, type of inoculated cells, duration of treatment, curcumin dose, administration mode, and type of curcumin formulation, the results’ heterogeneity was generally moderate or high. This is frequently seen in meta-analyses using results obtained from animal models [18], since in these types of studies, the sources of heterogeneity are hard to identify because the experimental conditions vary considerably between studies. However, animal models of diseases are fundamental in preclinical studies to clarify disease mechanisms and for testing new therapeutic approaches.

From the analysis of the studies’ quality rating, it can be concluded that the overall quality of the studies included in the present meta-analysis was good. Furthermore, the sensitivity analysis showed the reliability of the results obtained with the present meta-analysis. In the present meta-analysis, the publication bias was evaluated using funnel plots and Egger’s regression test. Considering the effect of curcumin alone on tumor growth, while Egger’s regression test showed the presence of a publication bias, funnel plot analysis did not reveal any discernible bias. Regarding the effect of combined curcumin and radiation, neither test revealed publication bias. Publication bias usually results from the fact that neutral studies frequently remain unreported or take longer to report when compared to those showing statistically significant findings [51]. This does not, however, invalidate the conclusions from the present meta-analysis aiming to evaluate the effect of curcumin alone, since the WSDM remained significant after performing the funnel plot analysis to correct for publication bias. However, it cannot be ruled out that other confounding factors of the studies’ design, like randomization, allocation concealment, and blinded outcome assessment, could also be a source of bias, as frequently occurs in animal research [52].

The available therapeutic options to treat gliomas, particularly glioblastoma, are not only limited but show modest efficacy. In fact, the standard treatment for glioblastoma, involving surgery followed by radiotherapy and chemotherapy, only results in a low overall survival, with a median survival of patients of 15 months after diagnosis [3]. The most common chemotherapeutic options, involving the use of alkylating agents, such as temozolomide, nitrosoureas, such as carmustine, and Avastin, only show some limited efficacy [4]. Therefore, the results obtained with the present meta-analysis, showing a decrease in tumor growth in glioma-bearing animal xenografts treated with curcumin, are interesting and raise the possibility of the potential use of curcumin, alone or in combination with other drugs or radiotherapy, to treat glioma patients and may encourage the start of human clinical trials. Clinical trials involving the use of curcumin in combination with other approved drugs have been described for other types of cancer, such as advanced pancreatic cancer, where curcumin was shown to improve the safety and efficacy of the chemotherapeutic drug gemcitabine in a phase II clinical trial [53].

Curcumin’s ability to reduce tumor volume involves a complex interplay of molecular mechanisms, including apoptosis induction [34,41] and necrosis [44], but can also involve other processes like autophagy [38] and inhibition of proliferation and angiogenesis [34]. The reduction in tumor volume attributed to curcumin’s action often involves a combination of these mechanisms, which collectively disrupt tumor cell survival, proliferation, and progression. The specific balance and dominance of these mechanisms may vary depending on the tumor type, cellular context, and other factors contributing to the overall reduction in tumor size. Research also suggests that curcumin exhibits promising effects against CSCs across various types of cancer. CSCs are a small population of cells within tumors that possess self-renewal capabilities and are implicated in tumor initiation, progression, and resistance to therapies. The available data from in vitro and in vivo studies indicate that curcumin holds the potential to target CSCs by disrupting their stemness properties, inducing differentiation, and inhibiting signaling pathways crucial for CSC maintenance across various cancers [11].

In vitro and in vivo studies show that curcumin may produce its anticancer action against glioma by reducing cell proliferation, migration, and invasion while increasing apoptosis and autophagy of glioma cells and decreasing angiogenesis (Figure 1) [6,11,12]. Curcumin may produce its anticancer effects by directly binding and inhibiting several protein targets such as (i) COX and lipoxygenase, consequently reducing inflammation; (ii) PKC, Src, and Erb2, leading to reduced cell proliferation; (iii) Bcl2, reducing cell survival; and (iv) P-12-LOX, decreasing angiogenesis [13]. Curcumin has also shown the ability to modulate several signaling pathways that are deregulated in glioma. Curcumin decreased the activity of transcription factors AP-1 and NF-κB [11,15]. The decrease in AP-1 activity may result in decreased invasion since this TF induces the expression of MMPs. A reduction in NF-κB may be a consequence of decreased PKB/Akt phosphorylation [15]—resulting in decreased NF-κB activation by Akt—and decreased proteasome degradation of the NF-kB inhibitor IκB [54], induced by curcumin. Decreased NF-κB activity—which is upregulated in glioma—induced by curcumin may result in decreased proliferation, cell survival, angiogenesis, inflammation, and increased apoptosis since this TF directly regulates the expression of genes involved in these processes [55]. Additionally, curcumin decreases the expression and phosphorylation of PI3K and mTOR, further contributing to reducing cell proliferation [12,16]. Curcumin has been shown to activate both the extrinsic and intrinsic apoptosis pathways by increasing levels of caspases 8, 9, and 3 and Bax while decreasing the expression of Bcl-2 [6,11]. It has also been shown to inhibit the cell cycle by increasing the expression of p53, p21, and p16 and decreasing the phosphorylation of Rb while decreasing the expression of cyclin D [6,11]. A decrease, induced by curcumin, in MMP, VEGF, and bFGF expression has been reported, with a consequent decrease in invasion and angiogenesis [6,11]. Curcumin also decreased epithelial–mesenchymal transition through inhibition of the Hedgehog pathway [6].

The findings of this study hold substantial promise for the clinical management of glioblastoma patients:

(i) Adjuvant therapy—curcumin emerges as a potential adjunct to conventional treatments, offering a supplementary path to augment the efficacy of current therapeutic regimens;
(ii) Minimization of side effects—the relatively low toxicity profile of curcumin, coupled with its demonstrated antitumor effects, suggests its potential to reduce treatment-related side effects and enhance overall patient tolerance to therapy;
(iii) Personalized treatment approaches—an exploration of curcumin’s effects opens possibilities for personalized medicine, where treatment strategies can be tailored based on individual patient profiles and molecular characteristics of their tumors;
(iv) Improved prognosis—integration of curcumin into treatment protocols may potentially lead to improved prognosis and enhanced survival rates for glioblastoma patients, particularly by addressing treatment resistance and disease recurrence.

Considering these clinical implications, it is recommended that rigorous clinical trials are conducted to validate the safety and efficacy of curcumin in glioblastoma patients. Emphasis should be placed on patient stratification and biomarker identification to identify responders effectively. Collaboration between oncologists, neurosurgeons, pharmacologists, and researchers is encouraged to expedite the translation of these findings into clinical practice. Efforts should be made to educate patients and healthcare providers about the potential benefits and limitations of curcumin as an adjunct therapy, fostering informed decision making and treatment discussions. Incorporating curcumin into the clinical armamentarium for glioblastoma has the potential to revolutionize treatment paradigms, offering new hope and avenues for improving patient outcomes.

Translating doses from preclinical studies to clinical use in humans requires careful consideration, as the efficacy and safety of a compound like curcumin can vary significantly between laboratory models and human subjects. Preclinical studies often use higher doses due to various factors, including different metabolic rates and body sizes between animals and humans, and to achieve observable effects in experimental settings. However, suggesting a specific clinical antitumor dose of curcumin based solely on preclinical doses might be challenging due to these differences [56]. Instead, a strategy called “allometric scaling” is commonly used to estimate a starting dose for human trials from preclinical data [57]. Therefore, while preclinical data might provide a starting point, determining an optimal clinical antitumor dose of curcumin requires rigorous clinical trials focused on safety, efficacy, and tolerability in human subjects. These trials help establish appropriate dosing regimens for potential therapeutic applications.

Furthermore, integrating our previous findings from studies on resveratrol [7] and cannabinoids [8] with the present results about curcumin for glioma treatment offers a comprehensive understanding and potential strategies to enhance the Stupp protocol [5]. Both resveratrol and cannabinoids exhibit antiglioma properties. Resveratrol, through its anti-inflammatory and antiproliferative actions, inhibits tumor growth [7]. Cannabinoids, like CBD and THC, have shown antiglioma effects by inducing apoptosis and inhibiting angiogenesis through cannabinoid receptor-mediated increase in ceramide production [8]. Interestingly, a previous study reported that blocking ERK1/2-mediated temozolomide and curcumin-induced protective autophagy with resveratrol improved temozolomide/curcumin efficacy in brain-implanted tumors [58].

The present study reviewed curcumin’s ability to modulate various pathways and mechanisms involved in glioblastoma growth inhibition, including inflammation, cell survival, and angiogenesis. Considering the findings from all three studies, a combined approach involving curcumin, resveratrol, and cannabinoids could present a multipronged attack on glioma cells. This combination might effectively target multiple pathways involved in tumor growth and progression. Integrating these natural compounds into the Stupp protocol could potentially augment its effectiveness. For example, administering these compounds alongside radiotherapy and temozolomide might enhance their antitumor effects. The combination of curcumin, resveratrol, and cannabinoids might complement the Stupp protocol by targeting pathways not adequately addressed by conventional therapies. This could improve treatment outcomes, reduce resistance, and minimize side effects. Tailoring treatments based on individual patient profiles and tumor characteristics could optimize the incorporation of these compounds into the Stupp protocol.

The broad-spectrum therapeutic effects of curcumin across various cancers suggest a common antitumor mechanism, but the nuances of its action may vary among different cancer types. The ability of curcumin to directly bind and inhibit several cellular proteins, such as proinflammatory COX and lipoxygenase, proliferation-inducing PKC, Src, Erb2, anti-apoptotic Bcl2, and pro-angiogenic P-12-LOX, which play a role in different types of malignant cells, might explain the broad antitumor action of curcumin described for different types of cancers [14,59]. Further research is needed to understand the specific nuances and optimize curcumin’s use as a potential universal antitumor agent across diverse cancer types.

The present work has some limitations related to the fact that the studies reviewed are primarily preclinical, limiting direct extrapolation to clinical settings. The effects observed in animal models may not entirely mirror responses in human patients. Variability in curcumin formulations, doses, treatment durations, and administration routes across studies could impact result consistency. Differences in protocols for assessing outcomes, such as tumor volume measurements, could introduce variability and affect result interpretation.

## 5. Conclusions

Curcumin was able to reduce tumor growth in rodent xenograft models of glioma. This antiglioma effect of curcumin was observed for both orthotopic and heterotopic animal models and for different types of glioma cells, administration routes, and curcumin formulations. The effect of curcumin was higher for longer treatment durations. Although the presence of publication bias was identified, this does not invalidate curcumin’s effectiveness against glioma. The findings obtained with the present meta-analysis are encouraging and might foster future investigation on the potential therapeutic use of curcumin against such devastating diseases as glioma.

Moving forward, several paths warrant exploration and integration into future studies: translation of preclinical findings into clinical trials to assess the safety and efficacy of curcumin as adjuvant therapy for glioblastoma patients; investigation into innovative delivery systems to improve bioavailability and ensure effective concentrations of curcumin within the central nervous system; further research into potential synergies between curcumin and standard treatments, aiming to overcome resistance and improve patient outcomes; and continued exploration of the precise molecular mechanisms by which curcumin exerts its antitumor effects in glioblastoma cells.

## Figures and Tables

**Table 2:** Subgroup analysis of the effects of curcumin on glioblastoma growth.

Variable	Fold Increase			
	No. of Studies	WSDM (95%CI)	*p*-Value	I^2^ (%)
Animal Model				
Heterotopic	13	−2.381 (−3.428 to −1.334)	<0.0001 *	81.98
Orthotopic	11	−2.580 (−5.846 to −0.686)	0.001 *	79.60
chi^2^ = 0.548; *p*-value = 0.459				
Cells				
Human glioblastoma	13	−1.594 (−2.480 to −0.708)	<0.0001 *	56.29
Moude glioma	2	−8.969 (−12.297 to −5.642)	<0.0001 *	69.90
Rat glioblastoma	2	−0.257 (−2.430 to 1.916)	0.818	0
Rat glioma	7	−2.281 (−3.481 to −1.081)	<0.0001 *	87.57
chi^2^ = 20.333; *p*-value < 0.0001 *				
Intervention duration (days)				
1–10	9	−1.893 (−3.035 to −0.751)	0.001 *	84.14
11–20	9	−1.219 (−1.398 to −0.039)	0.043 *	56.03
21–28	3	−2.529 (−7.484 to −2.933)	0.014 *	24.32
chi^2^ = 9.599; *p*-value = 0.022 *				
Administration mode				
Intraperitoneal	9	−3.138 (−2.079 to −0.200)	<0.0001 *	82.30
Intratumoral	3	−1.722 (−4.536 to −1.740)	0.109	6.77
Intravenous (via tail vein)	7	−2.338 (−3.853 to −0.823)	0.002 *	86.53
Oral	3	−0.421 (−2.582 to 1.740)	0.703	0
Peritumoral	1	−0.243 (−3.839 to 3.354)	0.895	0
chi^2^ = 5.786; *p*-value = 0.328				
Mean Daily Dose (mg/kg/day)				
0.0065–20	5	−1.456 (−3.094 to 0.181)	0.081	77.85
20.1–60	12	−2.268 (−3.405 to −1.130)	<0.0001 *	79.88
60.1–120	2	−1.178 (−3.805 to −1.488)	0.379	44.93
chi^2^ = 2.284; *p*-value = 0.516				
Total Dose (mg/kg)				
0.0065–200	5	−1.458 (−3.104 to 0.188)	0.083	77.85
200–900	10	−2.535 (−3.814 to −1.255)	<0.0001 *	83.01
901–1680	4	−1.209 (−1.293 to 0.196)	0.196	14.04
chi^2^ = 3.071; *p*-value = 0.381				
Formulation				
Free curcumin	13	−2.430 (−3.469 to −1.391)	<0.0001 *	74.53
Non-free curcumin	11	−1.736 (−2.842 to −0.631)	0.002 *	85.11
chi^2^ = 0.804; *p*-value = 0.370				

WSDM—weighted standardized difference in mean; CI—confidence interval; * indicates a significant result.

**Table 3:** Assessment of publication bias for the impact of curcumin administration on glioma growth.

Outcome	Egger’s Regression Test			
	95% CI	*t*	*p*-Value	df
Tumor volume (fold increase from day 1)	−5.273 to −1.365	3.522	0.022 *	22

CI—confidence interval; df—degrees of freedom; * indicates a significant result.

## Data Availability

Data will be available upon request to the corresponding author.

## Supplementary Materials

The following supporting information can be downloaded at: https://www.mdpi.com/article/10.3390/biomedicines12020268/s1, Table S1: Study quality scores; Table S2: Assessment of publication bias for the impact of effects of radiation alone against the administration of curcumin together with radiation; Figure S1: Results of sensitivity analysis for the meta-analysis of the effects of radiation alone against the administration of curcumin together with radiation [3,11]; Figure S2: Funnel plot of standard error by difference in means (publication bias tests) of the effects of radiation alone against the administration of curcumin together with radiation.
