# Supplementary material for: Action of Curcumin on Glioblastoma Growth: A Systematic Review with Meta-Analysis of Animal Model Studies
Source: Biomedicines. 2024 Jan 24;12(2):268. doi: 10.3390/biomedicines12020268 (PMC10886523; doi:10.3390/biomedicines12020268)
Supplement: Supplementary file 1 [file biomedicines-12-00268-s001.zip › biomedicines-2812672-supplementary.pdf]

Table S1: Study quality scores.

Study	Year	1	2	3	4	5	6	7	8	9	Quality score
Wang, et al [1]	2021	+	+	+	-	-	+	+	+	+	7
Xu, et al [2]	2020	+	+	+	-	-	-	+	+	-	5
Wang, et al A) [3]	2020	+	+	-	-	-	+	+	+	+	6
Wang, et al B) [4]	2020	+	+	+	-	-	+	+	+	+	7
He, et al 1) [5]	2020	+	+	-	-	-	-	+	-	-	3
He, et al 2) [5]	2020	+	+	-	-	-	-	+	-	-	3
Pan, et al [6]	2019	+	+	-	-	-	-	+	+	-	4
Jia, et al 1) [7]	2018	+	+	+	-	-	+	+	+	-	6
Jia, et al 2) [7]	2018	+	+	+	-	-	+	+	+	-	6
Li, et al 1) [8]	2017	+	+	+	-	-	+	+	-	-	5
Li, et al 2) [8]	2017	+	+	+	-	-	+	+	-	-	5
Li, et al 3) [8]	2017	+	+	+	-	-	+	+	-	-	5
Singh, et al [9]	2016	+	+	-	-	-	+	-	+	+	5
Orunoglu, et al 1) [10]	2017	+	+	+	-	-	+	-	+	-	5
Orunoglu, et al 2) [10]	2017	+	+	+	-	-	+	-	+	-	5
Orunoglu, et al 3) [10]	2017	+	+	+	-	-	+	-	+	-	5
Orunoglu, et al 4) [10]	2017	+	+	+	-	-	+	-	+	-	5
Meng, et al 1) [11]	2017	+	-	+	-	-	-	+	-	-	3
Meng, et al 2) [11]	2017	+	-	+	-	-	-	+	-	-	3
Zheng, et al 1) [12]	2016	+	+	+	-	-	+	+	+	-	6
Zheng, et al 2) [12]	2016	+	+	+	-	-	+	+	+	-	6
Yin, et al [13]	2014	+	+	+	-	-	+	-	+	-	5
Perry, et al [14]	2010	+	+	-	-	-	+	-	+	-	4
Aoki, et al [15]	2007	+	+	-	-	-	+	-	-	-	3

1) Peer-review publication;
2) Standardized number of tumor cells implanted;
3) Randomized allocation of tumor-bearing animals to treatment and control groups;
4) Blinded assessment of outcome;
5) Sample size calculation performed;
6) Compliance with animal welfare regulations;
7) Statement of potential conflicts of interest;
8) Reported the number of animals originally inoculated with tumor cells;
9) Reported the explanation of any treated animals excluded from analysis.

Table S2: Assessment of publication bias for the impact of effects of radiation alone against the administration of curcumin together with radiation.

Outcome	Egger's Regression Test			
	**95% CI**	***t***	***p*-Value**	**df**
Tumor volume (fold increase from day 1)	-56.240 to 41.701	1.886	1	0.310

CI - confidence interval; df - degrees of freedom.

Figure S1: Results of sensitivity analysis for the meta-analysis of the of effects of radiation alone against the administration of curcumin together with radiation [3,11].

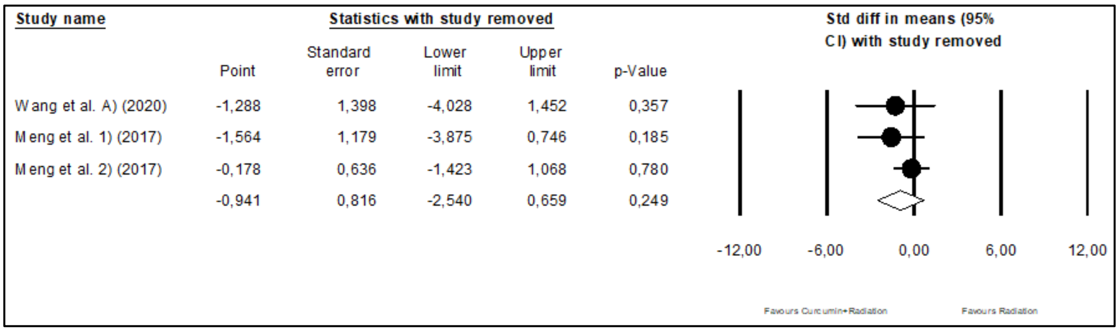

Figure S2: Funnel plot of standard error by difference in means (publication bias tests) of the of effects of radiation alone against the administration of curcumin together with radiation.

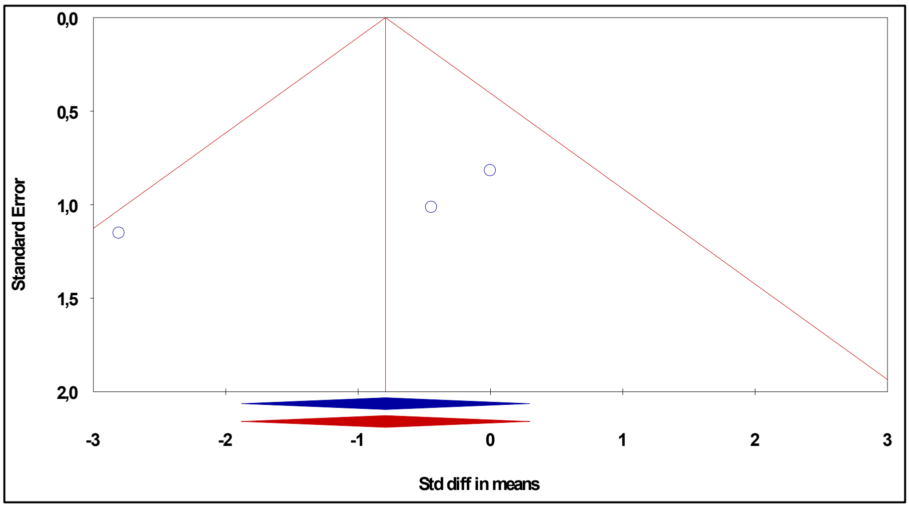

References

1. Wang, P.; Hao, X.; Li, X.; Yan, Y.; Tian, W.; Xiao, L.; Wang, Z.; Dong, J. Curcumin inhibits adverse psychological stress-induced proliferation and invasion of glioma cells via down-regulating the ERK/MAPK pathway. *J. Cell. Mol. Med.* **2021**, *25*, 7190–7203, doi: 10.1111/jcmm.16749.
2. Xu, M.; Li, G.; Zhang, H.; Chen, X.; Li, Y.; Yao, Q.; Xie, M. Sequential delivery of dual drugs with nanostructured lipid carriers for improving synergistic tumor treatment effect. *Drug Deliv.* **2020**, *27*, 983–995, doi: 10.1080/10717544.2020.1785581.
3. Wang, W.; Shen C.; Chien, Y.; Chang, W.; Tasi, C.; Lin, Y.; Hwang, J. Validation of enhancing effects of curcumin on radiotherapy with f98/fgt glioblastoma-bearing rat model. *Int. J. Mol. Sci.* **2020**, *21*, 4385, doi: 10.3390/ijms21124385.
4. Wang, Z.; Liu, F.; Liao, W.; Yu, L.; Hu, Z.; Li, M.; Xia, H. Curcumin suppresses glioblastoma cell proliferation by p-AKT/mTOR pathway and increases the PTEN expression. *Arch. Biochem. Biophys.* **2020**, *689*, 108412, doi: 10.1016/j.abb.2020.108412.
5. He, Y.; Wu, C.; Duan, J.; Miao, J.; Ren, H.; Liu, J. Anti-Glioma Effect with Targeting Therapy Using Folate Modified Nano-Micelles Delivery Curcumin. *J. Biomed. Nanotechnol.* **2020**, *16*, 1–13, doi: 10.1166/jbn.2020.2878.
6. Pan, J.X.; Chen, T.N.; Ma, K.; Wang, S.; Yang, C.Y.; Cui, G.Y. A negative feedback loop of H19/miR-675/VDR mediates therapeutic effect of cucurmin in the treatment of glioma. *J. Cell. Physiol.* **2020**, *235*, 2171–2182, doi: 10.1002/jcp.29127.
7. Jia, G.; Han, Y.; An, Y.; Ding, Y.; He, C.; Wang, X.; Tang, Q. NRP-1 targeted and cargo-loaded exosomes facilitate simultaneous imaging and therapy of glioma *in vitro* and *in vivo*. *Biomaterials* **2018**, *178*, 302–316, doi: 10.1016/j.biomaterials.2018.06.029.
8. Li, W.; Yang, W.; Liu, Y.; Chen, S.; Chin, S.; Qi, X.; Zhao, Y.; Liu, H.; Wang, J.; Mei, X.; Huang, P.; Xu, D. MicroRNA-378 enhances inhibitory effect of curcumin on glioblastoma. *Oncotarget* **2017**, *8*, 73938–73946, doi: 10.18632/oncotarget.17881.
9. Singh, A.; Kim, W.; Kim, Y.; Jeong, K.; Kang, C.S.; Kim, W.; Koh, J.; Mahajan, S.D.; Prasad, P.N.; Kim, S. Multifunctional

Photonics Nanoparticles for Crossing the Blood–Brain Barrier and Effecting Optically Trackable Brain Theranostics. *Adv. Funct. Mater.* **2016**, *26*, 7057–7066, doi: 10.1002/adfm.201602808.
10. Orunoglu, M.; Kaffashi, A.; Phelivan, S.B.; Sahin, S.; Söylemezoglu, F.; Karli, Oguz, K.K.; Mut, M. Effects of curcumin-loaded PLGA nanoparticles on the RG2 rat glioma model. *Mater. Sci. Eng. C* **2017**, *78*, 32–38, doi: 10.1016/j.msec.2017.03.292.
11. Meng, X.; Cai, J.; Liu, J.; Han, B.; Gao, F.; Gao, W.; Zhang, Y.; Zhang, J.; Zhao, Z.; Jiang, C. Curcumin increases efficiency of γ-irradiation in gliomas by inhibiting Hedgehog signaling pathway. *Cell Cycle* **2017**, *16*, 1181–1192, doi: 10.1080/15384101.2017.1320000.
12. Zheng, S.; Gao, X.; Liu, X.; Yu, T.; Zheng, T.; Wang, Y.; You, C. Biodegradable micelles enhance the antiglioma activity of curcumin *in vitro* and *in vivo*. *Int. J. Nanomedicine* **2016**, *11*, 2721–2736, doi: 10.2147/IJN.S102450.
13. Yin, H.; Zhou, Y.; Wen, C.; Zhou, C.; Zhang, W.; Hu, X.; Wang, L.; You, C.; Shao, J. Curcumin sensitizes glioblastoma to temozolomide by simultaneously generating ROS and disrupting AKT/mTOR signaling. *Oncol. Rep.* **2014**, *32*, 1610–1616, doi: 10.3892/or.2014.3342.
14. Perry, M.C.; Demeule, M.; Régina, A.; Moumdjian, R.; Béliveau, R. Curcumin inhibits tumor growth and angiogenesis in glioblastoma xenografts. *Mol. Nutr. Food Res.* **2010**, *54*, 1192–1201, doi: 10.1002/mnfr.200900277.
15. Aoki, H.; Takada, Y.; Kondo, S.; Sawaya, R.; Aggarwal, B.B.; Kondo, Y. Evidence that curcumin suppresses the growth of malignant gliomas *in vitro* and *in vivo* through induction of autophagy: Role of akt and extracellular signal-regulated kinase signaling pathways. *Mol. Pharmacol.* **2007**, *72*, 29–39, doi: 10.1124/mol.106.033167.
